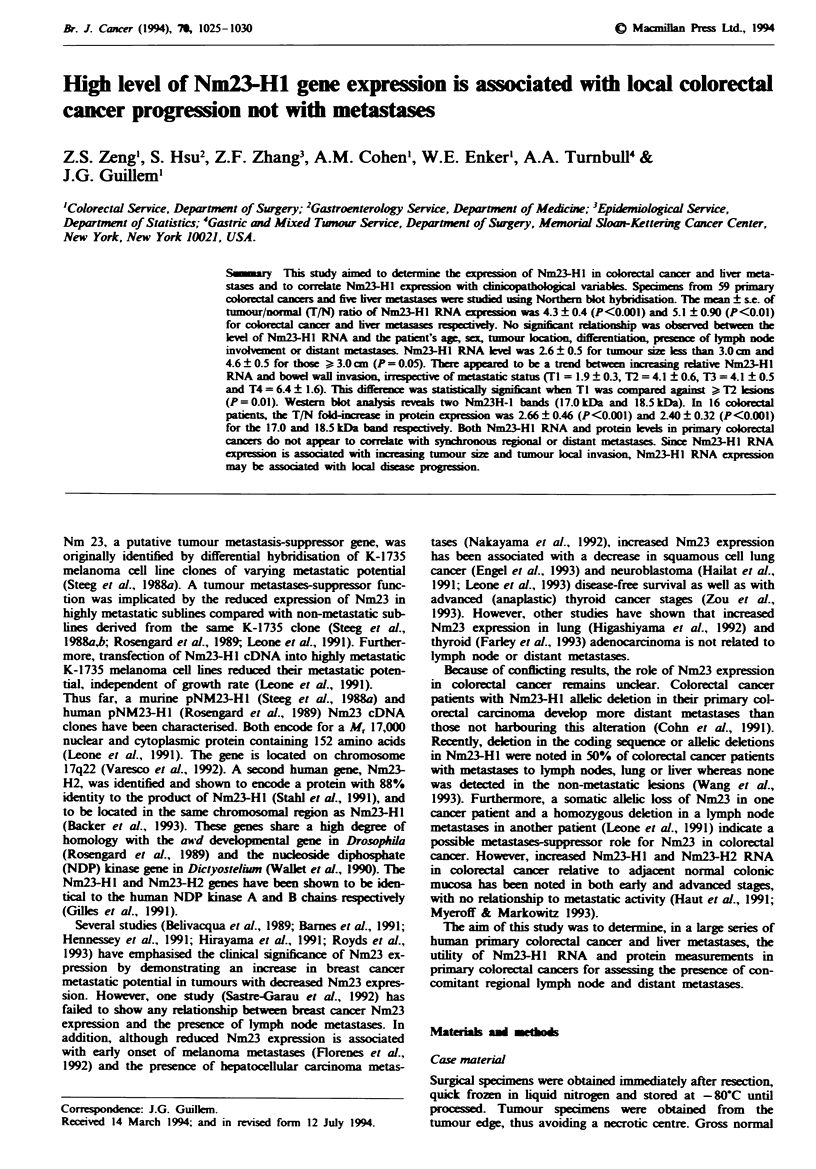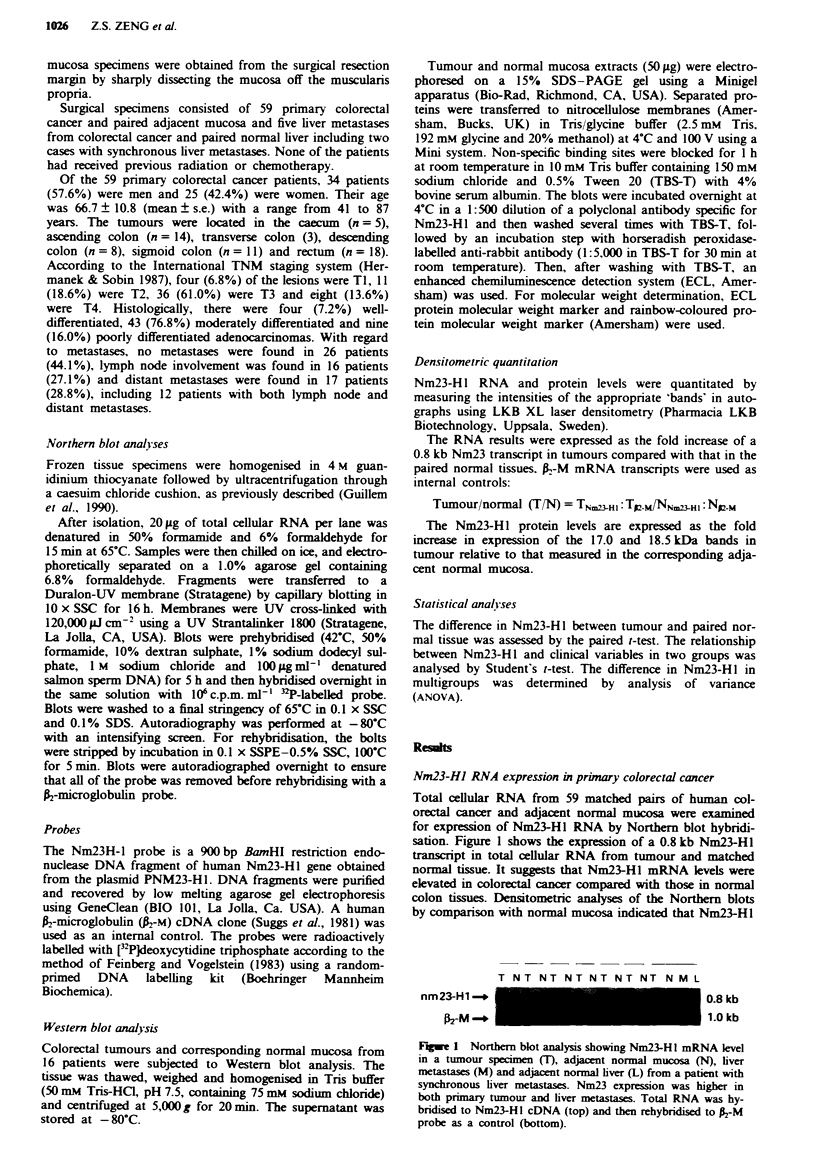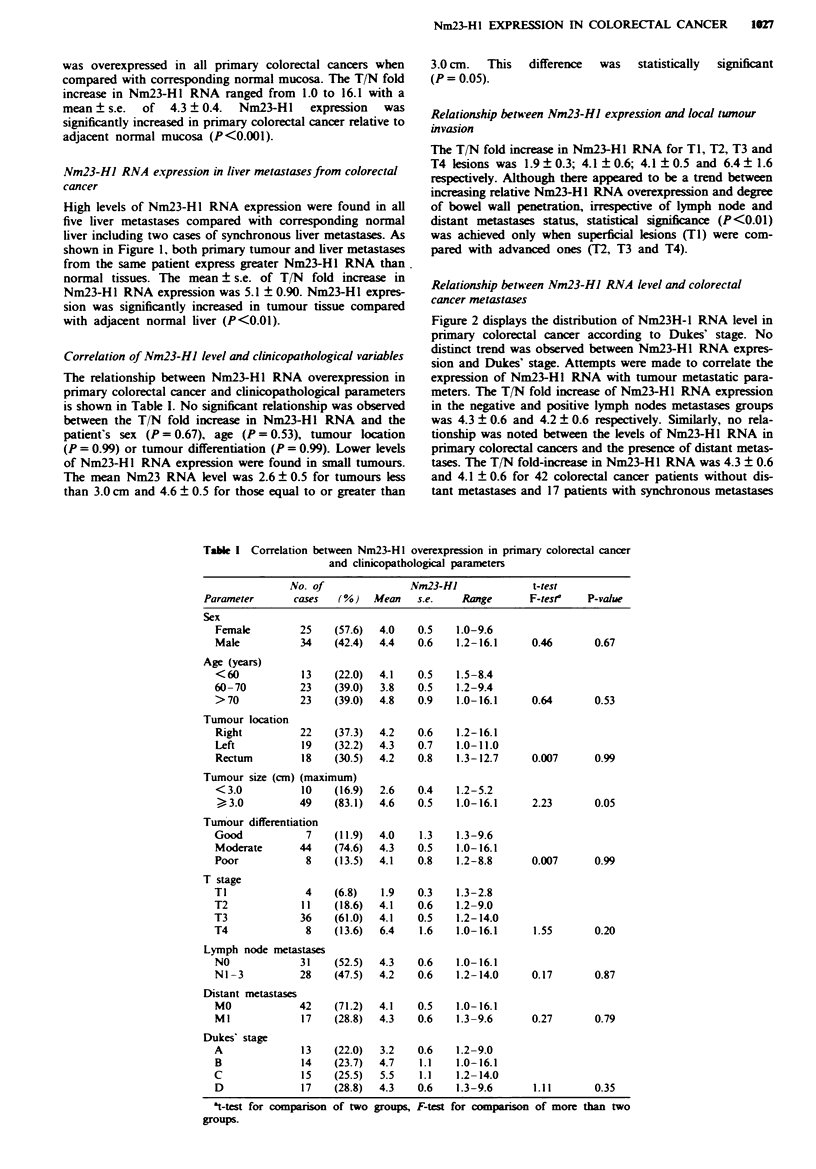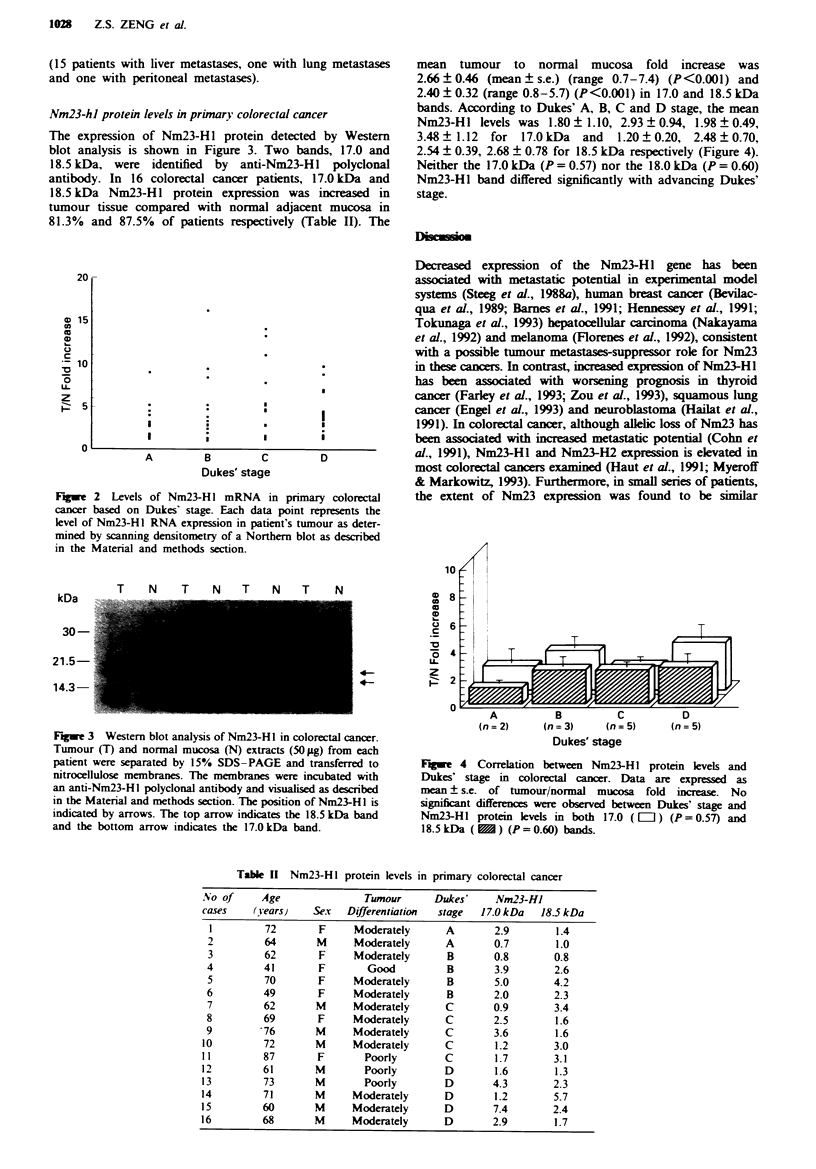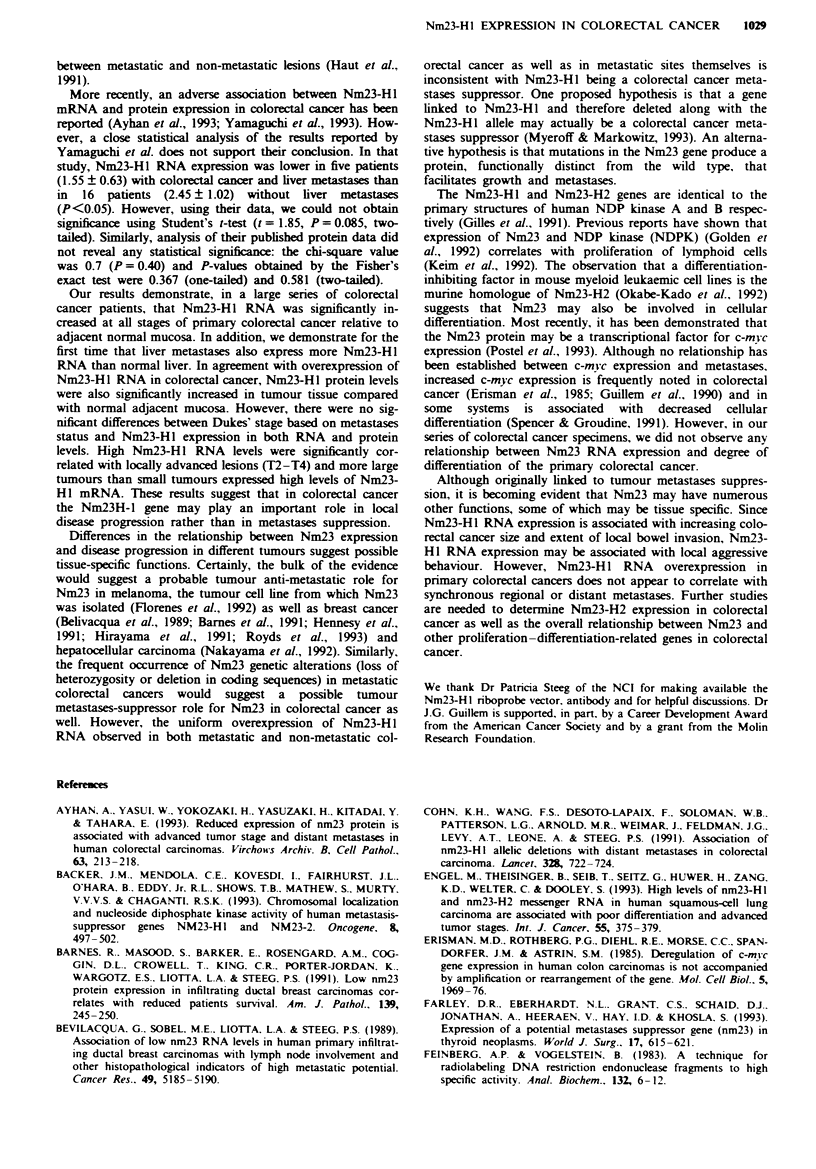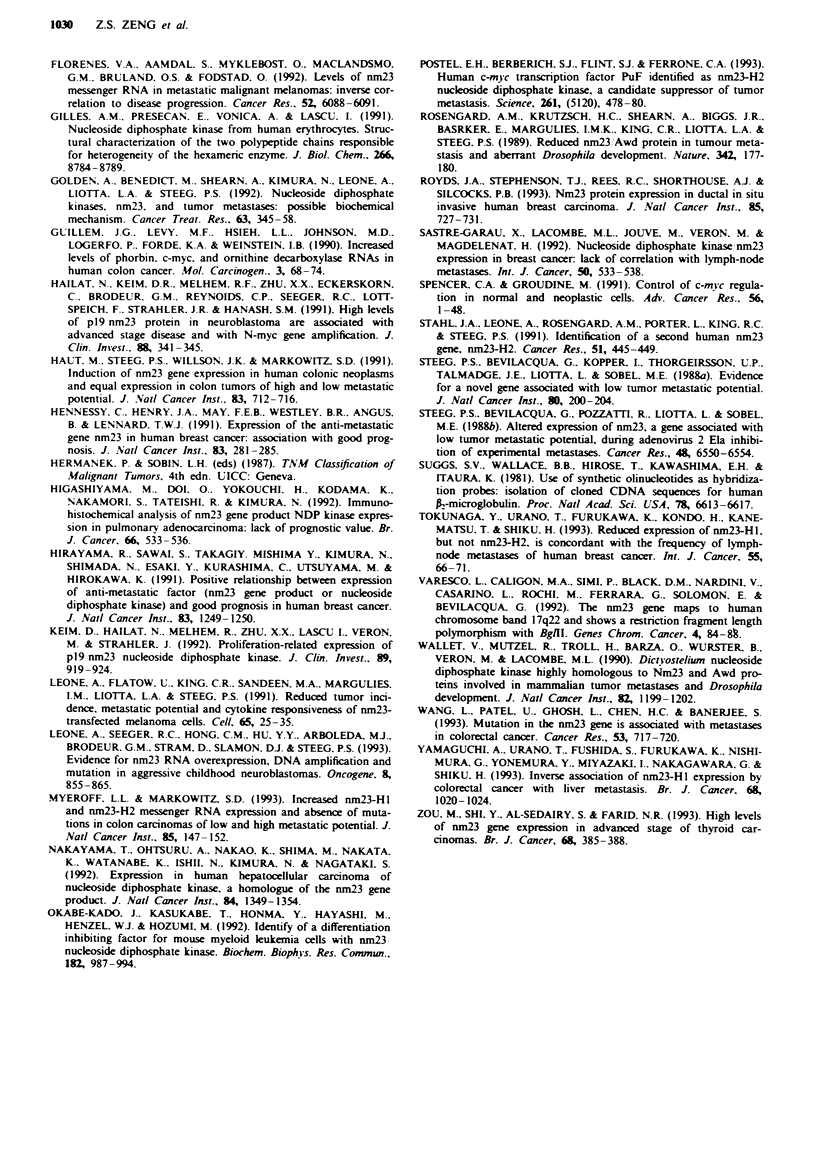# High level of Nm23-H1 gene expression is associated with local colorectal cancer progression not with metastases.

**DOI:** 10.1038/bjc.1994.442

**Published:** 1994-11

**Authors:** Z. S. Zeng, S. Hsu, Z. F. Zhang, A. M. Cohen, W. E. Enker, A. A. Turnbull, J. G. Guillem

**Affiliations:** Department of Surgery, Memorial Sloan-Kettering Cancer Center, New York, New York 10021.

## Abstract

**Images:**


Br. J. Cancer (1994), 70, 1025-1030

© Macmillan Press Ltd., 1994

### High level of Nm23-H1 gene expression is associated with local colorectal cancer progression not with metastases

Z.S. Zeng[1], S. Hsu[2], Z.F. Zhang[3], A.M. Cohen[1], W.E. Enker[1], A.A. Turnbull[4] & J.G. Guillem[1]

[1]*Colorectal Service, Department of Surgery;* [2]*Gastroenterology Service, Department of Medicine;* [3]*Epidemiological Service, Department of Statistics;* [4]*Gastric and Mixed Tumour Service, Department of Surgery, Memorial Sloan-Kettering Cancer Center, New York, New York 10021, USA.*

**Summary** This study aimed to determine the expression of Nm23-H1 in colorectal cancer and liver metastases and to correlate Nm23-H1 expression with clinicopathological variables. Specimens from 59 primary colorectal cancers and five liver metastases were studied using Northern blot hybridisation. The mean ± s.e. of tumour/normal (T/N) ratio of Nm23-H1 RNA expression was 4.3 ± 0.4 ($P<0.001$) and 5.1 ± 0.90 ($P<0.01$) for colorectal cancer and liver metasases respectively. No significant relationship was observed between the level of Nm23-H1 RNA and the patient's age, sex, tumour location, differentiation, presence of lymph node involvement or distant metastases. Nm23-H1 RNA level was 2.6 ± 0.5 for tumour size less than 3.0 cm and 4.6 ± 0.5 for those ⩾ 3.0 cm ($P=0.05$). There appeared to be a trend between increasing relative Nm23-H1 RNA and bowel wall invasion, irrespective of metastatic status (T1 = 1.9 ± 0.3, T2 = 4.1 ± 0.6, T3 = 4.1 ± 0.5 and T4 = 6.4 ± 1.6). This difference was statistically significant when T1 was compared against ⩾ T2 lesions ($P=0.01$). Western blot analysis reveals two Nm23H-1 bands (17.0 kDa and 18.5 kDa). In 16 colorectal patients, the T/N fold-increase in protein expression was 2.66 ± 0.46 ($P<0.001$) and 2.40 ± 0.32 ($P<0.001$) for the 17.0 and 18.5 kDa band respectively. Both Nm23-H1 RNA and protein levels in primary colorectal cancers do not appear to correlate with synchronous regional or distant metastases. Since Nm23-H1 RNA expression is associated with increasing tumour size and tumour local invasion, Nm23-H1 RNA expression may be associated with local disease progression.

Nm 23, a putative tumour metastasis-suppressor gene, was originally identified by differential hybridisation of K-1735 melanoma cell line clones of varying metastatic potential (Steeg *et al.*, 1988*a*). A tumour metastases-suppressor function was implicated by the reduced expression of Nm23 in highly metastatic sublines compared with non-metastatic sublines derived from the same K-1735 clone (Steeg *et al.*, 1988*a,b*; Rosengard *et al.*, 1989; Leone *et al.*, 1991). Furthermore, transfection of Nm23-H1 cDNA into highly metastatic K-1735 melanoma cell lines reduced their metastatic potential, independent of growth rate (Leone *et al.*, 1991).

Thus far, a murine pNM23-H1 (Steeg *et al.*, 1988*a*) and human pNM23-H1 (Rosengard *et al.*, 1989) Nm23 cDNA clones have been characterised. Both encode for a $M_r$ 17,000 nuclear and cytoplasmic protein containing 152 amino acids (Leone *et al.*, 1991). The gene is located on chromosome 17q22 (Varesco *et al.*, 1992). A second human gene, Nm23-H2, was identified and shown to encode a protein with 88% identity to the product of Nm23-H1 (Stahl *et al.*, 1991), and to be located in the same chromosomal region as Nm23-H1 (Backer *et al.*, 1993). These genes share a high degree of homology with the *awd* developmental gene in *Drosophila* (Rosengard *et al.*, 1989) and the nucleoside diphosphate (NDP) kinase gene in *Dictyostelium* (Wallet *et al.*, 1990). The Nm23-H1 and Nm23-H2 genes have been shown to be identical to the human NDP kinase A and B chains respectively (Gilles *et al.*, 1991).

Several studies (Belivacqua *et al.*, 1989; Barnes *et al.*, 1991; Hennessey *et al.*, 1991; Hirayama *et al.*, 1991; Royds *et al.*, 1993) have emphasised the clinical significance of Nm23 expression by demonstrating an increase in breast cancer metastatic potential in tumours with decreased Nm23 expression. However, one study (Sastre-Garau *et al.*, 1992) has failed to show any relationship between breast cancer Nm23 expression and the presence of lymph node metastases. In addition, although reduced Nm23 expression is associated with early onset of melanoma metastases (Florenes *et al.*, 1992) and the presence of hepatocellular carcinoma metastases (Nakayama *et al.*, 1992), increased Nm23 expression has been associated with a decrease in squamous cell lung cancer (Engel *et al.*, 1993) and neuroblastoma (Hailat *et al.*, 1991; Leone *et al.*, 1993) disease-free survival as well as with advanced (anaplastic) thyroid cancer stages (Zou *et al.*, 1993). However, other studies have shown that increased Nm23 expression in lung (Higashiyama *et al.*, 1992) and thyroid (Farley *et al.*, 1993) adenocarcinoma is not related to lymph node or distant metastases.

Because of conflicting results, the role of Nm23 expression in colorectal cancer remains unclear. Colorectal cancer patients with Nm23-H1 allelic deletion in their primary colorectal carcinoma develop more distant metastases than those not harbouring this alteration (Cohn *et al.*, 1991). Recently, deletion in the coding sequence or allelic deletions in Nm23-H1 were noted in 50% of colorectal cancer patients with metastases to lymph nodes, lung or liver whereas none was detected in the non-metastatic lesions (Wang *et al.*, 1993). Furthermore, a somatic allelic loss of Nm23 in one cancer patient and a homozygous deletion in a lymph node metastases in another patient (Leone *et al.*, 1991) indicate a possible metastases-suppressor role for Nm23 in colorectal cancer. However, increased Nm23-H1 and Nm23-H2 RNA in colorectal cancer relative to adjacent normal colonic mucosa has been noted in both early and advanced stages, with no relationship to metastatic activity (Haut *et al.*, 1991; Myeroff & Markowitz 1993).

The aim of this study was to determine, in a large series of human primary colorectal cancer and liver metastases, the utility of Nm23-H1 RNA and protein measurements in primary colorectal cancers for assessing the presence of concomitant regional lymph node and distant metastases.

### Materials and methods

### *Case material*

Surgical specimens were obtained immediately after resection, quick frozen in liquid nitrogen and stored at −80°C until processed. Tumour specimens were obtained from the tumour edge, thus avoiding a necrotic centre. Gross normal

Correspondence: J.G. Guillem.
Received 14 March 1994; and in revised form 12 July 1994.

mucosa specimens were obtained from the surgical resection margin by sharply dissecting the mucosa off the muscularis propria.

Surgical specimens consisted of 59 primary colorectal cancer and paired adjacent mucosa and five liver metastases from colorectal cancer and paired normal liver including two cases with synchronous liver metastases. None of the patients had received previous radiation or chemotherapy.

Of the 59 primary colorectal cancer patients, 34 patients (57.6%) were men and 25 (42.4%) were women. Their age was 66.7 ± 10.8 (mean ± s.e.) with a range from 41 to 87 years. The tumours were located in the caecum ($n = 5$), ascending colon ($n = 14$), transverse colon (3), descending colon ($n = 8$), sigmoid colon ($n = 11$) and rectum ($n = 18$). According to the International TNM staging system (Hermanek & Sobin 1987), four (6.8%) of the lesions were T1, 11 (18.6%) were T2, 36 (61.0%) were T3 and eight (13.6%) were T4. Histologically, there were four (7.2%) well-differentiated, 43 (76.8%) moderately differentiated and nine (16.0%) poorly differentiated adenocarcinomas. With regard to metastases, no metastases were found in 26 patients (44.1%), lymph node involvement was found in 16 patients (27.1%) and distant metastases were found in 17 patients (28.8%), including 12 patients with both lymph node and distant metastases.

*Northern blot analyses*

Frozen tissue specimens were homogenised in 4 M guanidinium thiocyanate followed by ultracentrifugation through a caesuim chloride cushion, as previously described (Guillem *et al.*, 1990).

After isolation, 20 μg of total cellular RNA per lane was denatured in 50% formamide and 6% formaldehyde for 15 min at 65°C. Samples were then chilled on ice, and electrophoretically separated on a 1.0% agarose gel containing 6.8% formaldehyde. Fragments were transferred to a Duralon-UV membrane (Stratagene) by capillary blotting in 10 × SSC for 16 h. Membranes were UV cross-linked with 120,000 μJ $cm^{-2}$ using a UV Strantalinker 1800 (Stratagene, La Jolla, CA, USA). Blots were prehybridised (42°C, 50% formamide, 10% dextran sulphate, 1% sodium dodecyl sulphate, 1 M sodium chloride and 100 μg $ml^{-1}$ denatured salmon sperm DNA) for 5 h and then hybridised overnight in the same solution with $10^6$ c.p.m. $ml^{-1}$ $^{32}P$-labelled probe. Blots were washed to a final stringency of 65°C in 0.1 × SSC and 0.1% SDS. Autoradiography was performed at −80°C with an intensifying screen. For rehybridisation, the bolts were stripped by incubation in 0.1 × SSPE–0.5% SSC, 100°C for 5 min. Blots were autoradiographed overnight to ensure that all of the probe was removed before rehybridising with a $\beta_2$-microglobulin probe.

*Probes*

The Nm23H-1 probe is a 900 bp *Bam*HI restriction endonuclease DNA fragment of human Nm23-H1 gene obtained from the plasmid PNM23-H1. DNA fragments were purified and recovered by low melting agarose gel electrophoresis using GeneClean (BIO 101, La Jolla, Ca. USA). A human $\beta_2$-microglobulin ($\beta_2$-M) cDNA clone (Suggs *et al.*, 1981) was used as an internal control. The probes were radioactively labelled with [$^{32}$P]deoxycytidine triphosphate according to the method of Feinberg and Vogelstein (1983) using a random-primed DNA labelling kit (Boehringer Mannheim Biochemica).

*Western blot analysis*

Colorectal tumours and corresponding normal mucosa from 16 patients were subjected to Western blot analysis. The tissue was thawed, weighed and homogenised in Tris buffer (50 mM Tris-HCl, pH 7.5, containing 75 mM sodium chloride) and centrifuged at 5,000 *g* for 20 min. The supernatant was stored at −80°C.

Tumour and normal mucosa extracts (50 μg) were electrophoresed on a 15% SDS–PAGE gel using a Minigel apparatus (Bio-Rad, Richmond, CA, USA). Separated proteins were transferred to nitrocellulose membranes (Amersham, Bucks, UK) in Tris/glycine buffer (2.5 mM Tris, 192 mM glycine and 20% methanol) at 4°C and 100 V using a Mini system. Non-specific binding sites were blocked for 1 h at room temperature in 10 mM Tris buffer containing 150 mM sodium chloride and 0.5% Tween 20 (TBS-T) with 4% bovine serum albumin. The blots were incubated overnight at 4°C in a 1:500 dilution of a polyclonal antibody specific for Nm23-H1 and then washed several times with TBS-T, followed by an incubation step with horseradish peroxidase-labelled anti-rabbit antibody (1:5,000 in TBS-T for 30 min at room temperature). Then, after washing with TBS-T, an enhanced chemiluminescence detection system (ECL, Amersham) was used. For molecular weight determination, ECL protein molecular weight marker and rainbow-coloured protein molecular weight marker (Amersham) were used.

*Densitometric quantitation*

Nm23-H1 RNA and protein levels were quantitated by measuring the intensities of the appropriate 'bands' in autographs using LKB XL laser densitometry (Pharmacia LKB Biotechnology, Uppsala, Sweden).

The RNA results were expressed as the fold increase of a 0.8 kb Nm23 transcript in tumours compared with that in the paired normal tissues, $\beta_2$-M mRNA transcripts were used as internal controls:

$$\text{Tumour/normal (T/N)} = T_{Nm23\text{-}H1} : T_{\beta2\text{-}M} / N_{Nm23\text{-}H1} : N_{\beta2\text{-}M}$$

The Nm23-H1 protein levels are expressed as the fold increase in expression of the 17.0 and 18.5 kDa bands in tumour relative to that measured in the corresponding adjacent normal mucosa.

*Statistical analyses*

The difference in Nm23-H1 between tumour and paired normal tissue was assessed by the paired *t*-test. The relationship between Nm23-H1 and clinical variables in two groups was analysed by Student's *t*-test. The difference in Nm23-H1 in multigroups was determined by analysis of variance (ANOVA).

### Results

*Nm23-H1 RNA expression in primary colorectal cancer*

Total cellular RNA from 59 matched pairs of human colorectal cancer and adjacent normal mucosa were examined for expression of Nm23-H1 RNA by Northern blot hybridisation. Figure 1 shows the expression of a 0.8 kb Nm23-H1 transcript in total cellular RNA from tumour and matched normal tissue. It suggests that Nm23-H1 mRNA levels were elevated in colorectal cancer compared with those in normal colon tissues. Densitometric analyses of the Northern blots by comparison with normal mucosa indicated that Nm23-H1

**Figure 1** Northern blot analysis showing Nm23-H1 mRNA level in a tumour specimen (T), adjacent normal mucosa (N), liver metastases (M) and adjacent normal liver (L) from a patient with synchronous liver metastases. Nm23 expression was higher in both primary tumour and liver metastases. Total RNA was hybridised to Nm23-H1 cDNA (top) and then rehybridised to $\beta_2$-M probe as a control (bottom).

was overexpressed in all primary colorectal cancers when compared with corresponding normal mucosa. The T/N fold increase in Nm23-H1 RNA ranged from 1.0 to 16.1 with a mean ± s.e. of 4.3 ± 0.4. Nm23-H1 expression was significantly increased in primary colorectal cancer relative to adjacent normal mucosa ($P<0.001$).

*Nm23-H1 RNA expression in liver metastases from colorectal cancer*

High levels of Nm23-H1 RNA expression were found in all five liver metastases compared with corresponding normal liver including two cases of synchronous liver metastases. As shown in Figure 1, both primary tumour and liver metastases from the same patient express greater Nm23-H1 RNA than normal tissues. The mean ± s.e. of T/N fold increase in Nm23-H1 RNA expression was 5.1 ± 0.90. Nm23-H1 expression was significantly increased in tumour tissue compared with adjacent normal liver ($P<0.01$).

*Correlation of Nm23-H1 level and clinicopathological variables*

The relationship between Nm23-H1 RNA overexpression in primary colorectal cancer and clinicopathological parameters is shown in Table I. No significant relationship was observed between the T/N fold increase in Nm23-H1 RNA and the patient's sex ($P = 0.67$), age ($P = 0.53$), tumour location ($P = 0.99$) or tumour differentiation ($P = 0.99$). Lower levels of Nm23-H1 RNA expression were found in small tumours. The mean Nm23 RNA level was 2.6 ± 0.5 for tumours less than 3.0 cm and 4.6 ± 0.5 for those equal to or greater than 3.0 cm. This difference was statistically significant ($P = 0.05$).

*Relationship between Nm23-H1 expression and local tumour invasion*

The T/N fold increase in Nm23-H1 RNA for T1, T2, T3 and T4 lesions was 1.9 ± 0.3; 4.1 ± 0.6; 4.1 ± 0.5 and 6.4 ± 1.6 respectively. Although there appeared to be a trend between increasing relative Nm23-H1 RNA overexpression and degree of bowel wall penetration, irrespective of lymph node and distant metastases status, statistical significance ($P<0.01$) was achieved only when superficial lesions (T1) were compared with advanced ones (T2, T3 and T4).

*Relationship between Nm23-H1 RNA level and colorectal cancer metastases*

Figure 2 displays the distribution of Nm23H-1 RNA level in primary colorectal cancer according to Dukes' stage. No distinct trend was observed between Nm23-H1 RNA expression and Dukes' stage. Attempts were made to correlate the expression of Nm23-H1 RNA with tumour metastatic parameters. The T/N fold increase of Nm23-H1 RNA expression in the negative and positive lymph nodes metastases groups was 4.3 ± 0.6 and 4.2 ± 0.6 respectively. Similarly, no relationship was noted between the levels of Nm23-H1 RNA in primary colorectal cancers and the presence of distant metastases. The T/N fold-increase in Nm23-H1 RNA was 4.3 ± 0.6 and 4.1 ± 0.6 for 42 colorectal cancer patients without distant metastases and 17 patients with synchronous metastases

**Table I** Correlation between Nm23-H1 overexpression in primary colorectal cancer and clinicopathological parameters

| | No. of | | | Nm23-H1 | | t-test | |
|---|---|---|---|---|---|---|---|
| *Parameter* | *cases* | *(%)* | *Mean* | *s.e.* | *Range* | F-*test*[a] | P-*value* |
| Sex | | | | | | | |
| Female | 25 | (57.6) | 4.0 | 0.5 | 1.0–9.6 | | |
| Male | 34 | (42.4) | 4.4 | 0.6 | 1.2–16.1 | 0.46 | 0.67 |
| Age (years) | | | | | | | |
| <60 | 13 | (22.0) | 4.1 | 0.5 | 1.5–8.4 | | |
| 60–70 | 23 | (39.0) | 3.8 | 0.5 | 1.2–9.4 | | |
| >70 | 23 | (39.0) | 4.8 | 0.9 | 1.0–16.1 | 0.64 | 0.53 |
| Tumour location | | | | | | | |
| Right | 22 | (37.3) | 4.2 | 0.6 | 1.2–16.1 | | |
| Left | 19 | (32.2) | 4.3 | 0.7 | 1.0–11.0 | | |
| Rectum | 18 | (30.5) | 4.2 | 0.8 | 1.3–12.7 | 0.007 | 0.99 |
| Tumour size (cm) (maximum) | | | | | | | |
| <3.0 | 10 | (16.9) | 2.6 | 0.4 | 1.2–5.2 | | |
| ⩾3.0 | 49 | (83.1) | 4.6 | 0.5 | 1.0–16.1 | 2.23 | 0.05 |
| Tumour differentiation | | | | | | | |
| Good | 7 | (11.9) | 4.0 | 1.3 | 1.3–9.6 | | |
| Moderate | 44 | (74.6) | 4.3 | 0.5 | 1.0–16.1 | | |
| Poor | 8 | (13.5) | 4.1 | 0.8 | 1.2–8.8 | 0.007 | 0.99 |
| T stage | | | | | | | |
| T1 | 4 | (6.8) | 1.9 | 0.3 | 1.3–2.8 | | |
| T2 | 11 | (18.6) | 4.1 | 0.6 | 1.2–9.0 | | |
| T3 | 36 | (61.0) | 4.1 | 0.5 | 1.2–14.0 | | |
| T4 | 8 | (13.6) | 6.4 | 1.6 | 1.0–16.1 | 1.55 | 0.20 |
| Lymph node metastases | | | | | | | |
| N0 | 31 | (52.5) | 4.3 | 0.6 | 1.0–16.1 | | |
| N1–3 | 28 | (47.5) | 4.2 | 0.6 | 1.2–14.0 | 0.17 | 0.87 |
| Distant metastases | | | | | | | |
| M0 | 42 | (71.2) | 4.1 | 0.5 | 1.0–16.1 | | |
| M1 | 17 | (28.8) | 4.3 | 0.6 | 1.3–9.6 | 0.27 | 0.79 |
| Dukes' stage | | | | | | | |
| A | 13 | (22.0) | 3.2 | 0.6 | 1.2–9.0 | | |
| B | 14 | (23.7) | 4.7 | 1.1 | 1.0–16.1 | | |
| C | 15 | (25.5) | 5.5 | 1.1 | 1.2–14.0 | | |
| D | 17 | (28.8) | 4.3 | 0.6 | 1.3–9.6 | 1.11 | 0.35 |

[a]t-test for comparison of two groups, *F*-test for comparison of more than two groups.

(15 patients with liver metastases, one with lung metastases and one with peritoneal metastases).

### *Nm23-h1 protein levels in primary colorectal cancer*

The expression of Nm23-H1 protein detected by Western blot analysis is shown in Figure 3. Two bands, 17.0 and 18.5 kDa, were identified by anti-Nm23-H1 polyclonal antibody. In 16 colorectal cancer patients, 17.0 kDa and 18.5 kDa Nm23-H1 protein expression was increased in tumour tissue compared with normal adjacent mucosa in 81.3% and 87.5% of patients respectively (Table II). The mean tumour to normal mucosa fold increase was 2.66 ± 0.46 (mean ± s.e.) (range 0.7–7.4) ($P<0.001$) and 2.40 ± 0.32 (range 0.8–5.7) ($P<0.001$) in 17.0 and 18.5 kDa bands. According to Dukes' A, B, C and D stage, the mean Nm23-H1 levels was 1.80 ± 1.10, 2.93 ± 0.94, 1.98 ± 0.49, 3.48 ± 1.12 for 17.0 kDa and 1.20 ± 0.20, 2.48 ± 0.70, 2.54 ± 0.39, 2.68 ± 0.78 for 18.5 kDa respectively (Figure 4). Neither the 17.0 kDa ($P = 0.57$) nor the 18.0 kDa ($P = 0.60$) Nm23-H1 band differed significantly with advancing Dukes' stage.

**Figure 2** Levels of Nm23-H1 mRNA in primary colorectal cancer based on Dukes' stage. Each data point represents the level of Nm23-H1 RNA expression in patient's tumour as determined by scanning densitometry of a Northern blot as described in the Material and methods section.

**Figure 3** Western blot analysis of Nm23-H1 in colorectal cancer. Tumour (T) and normal mucosa (N) extracts (50 µg) from each patient were separated by 15% SDS-PAGE and transferred to nitrocellulose membranes. The membranes were incubated with an anti-Nm23-H1 polyclonal antibody and visualised as described in the Material and methods section. The position of Nm23-H1 is indicated by arrows. The top arrow indicates the 18.5 kDa band and the bottom arrow indicates the 17.0 kDa band.

### Discussion

Decreased expression of the Nm23-H1 gene has been associated with metastatic potential in experimental model systems (Steeg et al., 1988a), human breast cancer (Bevilacqua et al., 1989; Barnes et al., 1991; Hennessey et al., 1991; Tokunaga et al., 1993) hepatocellular carcinoma (Nakayama et al., 1992) and melanoma (Florenes et al., 1992), consistent with a possible tumour metastases-suppressor role for Nm23 in these cancers. In contrast, increased expression of Nm23-H1 has been associated with worsening prognosis in thyroid cancer (Farley et al., 1993; Zou et al., 1993), squamous lung cancer (Engel et al., 1993) and neuroblastoma (Hailat et al., 1991). In colorectal cancer, although allelic loss of Nm23 has been associated with increased metastatic potential (Cohn et al., 1991), Nm23-H1 and Nm23-H2 expression is elevated in most colorectal cancers examined (Haut et al., 1991; Myeroff & Markowitz, 1993). Furthermore, in small series of patients, the extent of Nm23 expression was found to be similar

**Figure 4** Correlation between Nm23-H1 protein levels and Dukes' stage in colorectal cancer. Data are expressed as mean ± s.e. of tumour/normal mucosa fold increase. No significant differences were observed between Dukes' stage and Nm23-H1 protein levels in both 17.0 (□) ($P = 0.57$) and 18.5 kDa (▨) ($P = 0.60$) bands.

**Table II** Nm23-H1 protein levels in primary colorectal cancer

| *No of cases* | *Age (years)* | *Sex* | *Tumour Differentiation* | *Dukes' stage* | *Nm23-H1 17.0 kDa* | *Nm23-H1 18.5 kDa* |
|---|---|---|---|---|---|---|
| 1 | 72 | F | Moderately | A | 2.9 | 1.4 |
| 2 | 64 | M | Moderately | A | 0.7 | 1.0 |
| 3 | 62 | F | Moderately | B | 0.8 | 0.8 |
| 4 | 41 | F | Good | B | 3.9 | 2.6 |
| 5 | 70 | F | Moderately | B | 5.0 | 4.2 |
| 6 | 49 | F | Moderately | B | 2.0 | 2.3 |
| 7 | 62 | M | Moderately | C | 0.9 | 3.4 |
| 8 | 69 | F | Moderately | C | 2.5 | 1.6 |
| 9 | 76 | M | Moderately | C | 3.6 | 1.6 |
| 10 | 72 | M | Moderately | C | 1.2 | 3.0 |
| 11 | 87 | F | Poorly | C | 1.7 | 3.1 |
| 12 | 61 | M | Poorly | D | 1.6 | 1.3 |
| 13 | 73 | M | Poorly | D | 4.3 | 2.3 |
| 14 | 71 | M | Moderately | D | 1.2 | 5.7 |
| 15 | 60 | M | Moderately | D | 7.4 | 2.4 |
| 16 | 68 | M | Moderately | D | 2.9 | 1.7 |

between metastatic and non-metastatic lesions (Haut *et al.*, 1991).

More recently, an adverse association between Nm23-H1 mRNA and protein expression in colorectal cancer has been reported (Ayhan *et al.*, 1993; Yamaguchi *et al.*, 1993). However, a close statistical analysis of the results reported by Yamaguchi *et al.* does not support their conclusion. In that study, Nm23-H1 RNA expression was lower in five patients ($1.55 \pm 0.63$) with colorectal cancer and liver metastases than in 16 patients ($2.45 \pm 1.02$) without liver metastases ($P<0.05$). However, using their data, we could not obtain significance using Student's *t*-test ($t = 1.85$, $P = 0.085$, two-tailed). Similarly, analysis of their published protein data did not reveal any statistical significance: the chi-square value was 0.7 ($P = 0.40$) and *P*-values obtained by the Fisher's exact test were 0.367 (one-tailed) and 0.581 (two-tailed).

Our results demonstrate, in a large series of colorectal cancer patients, that Nm23-H1 RNA was significantly increased at all stages of primary colorectal cancer relative to adjacent normal mucosa. In addition, we demonstrate for the first time that liver metastases also express more Nm23-H1 RNA than normal liver. In agreement with overexpression of Nm23-H1 RNA in colorectal cancer, Nm23-H1 protein levels were also significantly increased in tumour tissue compared with normal adjacent mucosa. However, there were no significant differences between Dukes' stage based on metastases status and Nm23-H1 expression in both RNA and protein levels. High Nm23-H1 RNA levels were significantly correlated with locally advanced lesions (T2–T4) and more large tumours than small tumours expressed high levels of Nm23-H1 mRNA. These results suggest that in colorectal cancer the Nm23H-1 gene may play an important role in local disease progression rather than in metastases suppression.

Differences in the relationship between Nm23 expression and disease progression in different tumours suggest possible tissue-specific functions. Certainly, the bulk of the evidence would suggest a probable tumour anti-metastatic role for Nm23 in melanoma, the tumour cell line from which Nm23 was isolated (Florenes *et al.*, 1992) as well as breast cancer (Belivacqua *et al.*, 1989; Barnes *et al.*, 1991; Hennesy *et al.*, 1991; Hirayama *et al.*, 1991; Royds *et al.*, 1993) and hepatocellular carcinoma (Nakayama *et al.*, 1992). Similarly, the frequent occurrence of Nm23 genetic alterations (loss of heterozygosity or deletion in coding sequences) in metastatic colorectal cancers would suggest a possible tumour metastases-suppressor role for Nm23 in colorectal cancer as well. However, the uniform overexpression of Nm23-H1 RNA observed in both metastatic and non-metastatic colorectal cancer as well as in metastatic sites themselves is inconsistent with Nm23-H1 being a colorectal cancer metastases suppressor. One proposed hypothesis is that a gene linked to Nm23-H1 and therefore deleted along with the Nm23-H1 allele may actually be a colorectal cancer metastases suppressor (Myeroff & Markowitz, 1993). An alternative hypothesis is that mutations in the Nm23 gene produce a protein, functionally distinct from the wild type, that facilitates growth and metastases.

The Nm23-H1 and Nm23-H2 genes are identical to the primary structures of human NDP kinase A and B respectively (Gilles *et al.*, 1991). Previous reports have shown that expression of Nm23 and NDP kinase (NDPK) (Golden *et al.*, 1992) correlates with proliferation of lymphoid cells (Keim *et al.*, 1992). The observation that a differentiation-inhibiting factor in mouse myeloid leukaemic cell lines is the murine homologue of Nm23-H2 (Okabe-Kado *et al.*, 1992) suggests that Nm23 may also be involved in cellular differentiation. Most recently, it has been demonstrated that the Nm23 protein may be a transcriptional factor for c-*myc* expression (Postel *et al.*, 1993). Although no relationship has been established between c-*myc* expression and metastases, increased c-*myc* expression is frequently noted in colorectal cancer (Erisman *et al.*, 1985; Guillem *et al.*, 1990) and in some systems is associated with decreased cellular differentiation (Spencer & Groudine, 1991). However, in our series of colorectal cancer specimens, we did not observe any relationship between Nm23 RNA expression and degree of differentiation of the primary colorectal cancer.

Although originally linked to tumour metastases suppression, it is becoming evident that Nm23 may have numerous other functions, some of which may be tissue specific. Since Nm23-H1 RNA expression is associated with increasing colorectal cancer size and extent of local bowel invasion, Nm23-H1 RNA expression may be associated with local aggressive behaviour. However, Nm23-H1 RNA overexpression in primary colorectal cancers does not appear to correlate with synchronous regional or distant metastases. Further studies are needed to determine Nm23-H2 expression in colorectal cancer as well as the overall relationship between Nm23 and other proliferation–differentiation-related genes in colorectal cancer.

We thank Dr Patricia Steeg of the NCI for making available the Nm23-H1 riboprobe vector, antibody and for helpful discussions. Dr J.G. Guillem is supported, in part, by a Career Development Award from the American Cancer Society and by a grant from the Molin Research Foundation.

### References

AYHAN, A., YASUI, W., YOKOZAKI, H., YASUZAKI, H., KITADAI, Y. & TAHARA, E. (1993). Reduced expression of nm23 protein is associated with advanced tumor stage and distant metastases in human colorectal carcinomas. *Virchows Archiv. B, Cell Pathol.*, **63**, 213–218.

BACKER, J.M., MENDOLA, C.E., KOVESDI, I., FAIRHURST, J.L., O'HARA, B., EDDY, Jr, R.L., SHOWS, T.B., MATHEW, S., MURTY, V.V.V.S. & CHAGANTI, R.S.K. (1993). Chromosomal localization and nucleoside diphosphate kinase activity of human metastasis-suppressor genes NM23-H1 and NM23-2. *Oncogene*, **8**, 497–502.

BARNES, R., MASOOD, S., BARKER, E., ROSENGARD, A.M., COGGIN, D.L., CROWELL, T., KING, C.R., PORTER-JORDAN, K., WARGOTZ, E.S., LIOTTA, L.A. & STEEG, P.S. (1991). Low nm23 protein expression in infiltrating ductal breast carcinomas correlates with reduced patients survival. *Am. J. Pathol.*, **139**, 245–250.

BEVILACQUA, G., SOBEL, M.E., LIOTTA, L.A. & STEEG, P.S. (1989). Association of low nm23 RNA levels in human primary infiltrating ductal breast carcinomas with lymph node involvement and other histopathological indicators of high metastatic potential. *Cancer Res.*, **49**, 5185–5190.

COHN, K.H., WANG, F.S., DESOTO-LAPAIX, F., SOLOMAN, W.B., PATTERSON, L.G., ARNOLD, M.R., WEIMAR, J., FELDMAN, J.G., LEVY, A.T., LEONE, A. & STEEG, P.S. (1991). Association of nm23-H1 allelic deletions with distant metastases in colorectal carcinoma. *Lancet*, **328**, 722–724.

ENGEL, M., THEISINGER, B., SEIB, T., SEITZ, G., HUWER, H., ZANG, K.D., WELTER, C. & DOOLEY, S. (1993). High levels of nm23-H1 and nm23-H2 messenger RNA in human squamous-cell lung carcinoma are associated with poor differentiation and advanced tumor stages. *Int. J. Cancer*, **55**, 375–379.

ERISMAN, M.D., ROTHBERG, P.G., DIEHL, R.E., MORSE, C.C., SPANDORFER, J.M. & ASTRIN, S.M. (1985). Deregulation of c-*myc* gene expression in human colon carcinomas is not accompanied by amplification or rearrangement of the gene. *Mol. Cell Biol.*, **5**, 1969–76.

FARLEY, D.R., EBERHARDT, N.L., GRANT, C.S., SCHAID, D.J., JONATHAN, A., HEERAEN, V., HAY, I.D. & KHOSLA, S. (1993). Expression of a potential metastases suppressor gene (nm23) in thyroid neoplasms. *World J. Surg.*, **17**, 615–621.

FEINBERG, A.P. & VOGELSTEIN, B. (1983). A technique for radiolabeling DNA restriction endonuclease fragments to high specific activity. *Anal. Biochem.*, **132**, 6–12.

FLORENES, V.A., AAMDAL, S., MYKLEBOST, O., MACLANDSMO, G.M., BRULAND, O.S. & FODSTAD, O. (1992). Levels of nm23 messenger RNA in metastatic malignant melanomas: inverse correlation to disease progression. *Cancer Res.*, **52**, 6088-6091.

GILLES, A.M., PRESECAN, E., VONICA, A. & LASCU, I. (1991). Nucleoside diphosphate kinase from human erythrocytes. Structural characterization of the two polypeptide chains responsible for heterogeneity of the hexameric enzyme. *J. Biol. Chem.*, **266**, 8784-8789.

GOLDEN, A., BENEDICT, M., SHEARN, A., KIMURA, N., LEONE, A., LIOTTA, L.A. & STEEG, P.S. (1992). Nucleoside diphosphate kinases, nm23, and tumor metastases: possible biochemical mechanism. *Cancer Treat. Res.*, **63**, 345-58.

GUILLEM, J.G., LEVY, M.F., HSIEH, L.L., JOHNSON, M.D., LOGERFO, P., FORDE, K.A. & WEINSTEIN, I.B. (1990). Increased levels of phorbin, c-myc, and ornithine decarboxylase RNAs in human colon cancer. *Mol. Carcinogen.*, **3**, 68-74.

HAILAT, N., KEIM, D.R., MELHEM, R.F., ZHU, X.X., ECKERSKORN, C., BRODEUR, G.M., REYNOIDS, C.P., SEEGER, R.C., LOTTSPEICH, F., STRAHLER, J.R. & HANASH, S.M. (1991). High levels of p19/nm23 protein in neuroblastoma are associated with advanced stage disease and with N-myc gene amplification. *J. Clin. Invest.*, **88**, 341-345.

HAUT, M., STEEG, P.S., WILLSON, J.K. & MARKOWITZ, S.D. (1991). Induction of nm23 gene expression in human colonic neoplasms and equal expression in colon tumors of high and low metastatic potential. *J. Natl Cancer Inst.*, **83**, 712-716.

HENNESSY, C., HENRY, J.A., MAY, F.E.B., WESTLEY, B.R., ANGUS, B. & LENNARD, T.W.J. (1991). Expression of the anti-metastatic gene nm23 in human breast cancer: association with good prognosis. *J. Natl Cancer Inst.*, **83**, 281-285.

HERMANEK, P. & SOBIN, L.H. (eds) (1987). *TNM Classification of Malignant Tumors*, 4th edn. UICC: Geneva.

HIGASHIYAMA, M., DOI, O., YOKOUCHI, H., KODAMA, K., NAKAMORI, S., TATEISHI, R. & KIMURA, N. (1992). Immunohistochemical analysis of nm23 gene product/NDP kinase expression in pulmonary adenocarcinoma: lack of prognostic value. *Br. J. Cancer*, **66**, 533-536.

HIRAYAMA, R., SAWAI, S., TAKAGIY, MISHIMA Y., KIMURA, N., SHIMADA, N., ESAKI, Y., KURASHIMA, C., UTSUYAMA, M. & HIROKAWA, K. (1991). Positive relationship between expression of anti-metastatic factor (nm23 gene product or nucleoside diphosphate kinase) and good prognosis in human breast cancer. *J. Natl Cancer Inst.*, **83**, 1249-1250.

KEIM, D., HAILAT, N., MELHEM, R., ZHU, X.X., LASCU I., VERON, M. & STRAHLER, J. (1992). Proliferation-related expression of p19/nm23 nucleoside diphosphate kinase. *J. Clin. Invest.*, **89**, 919-924.

LEONE, A., FLATOW, U., KING, C.R., SANDEEN, M.A., MARGULIES, I.M., LIOTTA, L.A. & STEEG, P.S. (1991). Reduced tumor incidence, metastatic potential and cytokine responsiveness of nm23-transfected melanoma cells. *Cell*, **65**, 25-35.

LEONE, A., SEEGER, R.C., HONG, C.M., HU, Y.Y., ARBOLEDA, M.J., BRODEUR, G.M., STRAM, D., SLAMON, D.J. & STEEG, P.S. (1993). Evidence for nm23 RNA overexpression, DNA amplification and mutation in aggressive childhood neuroblastomas. *Oncogene*, **8**, 855-865.

MYEROFF, L.L. & MARKOWITZ, S.D. (1993). Increased nm23-H1 and nm23-H2 messenger RNA expression and absence of mutations in colon carcinomas of low and high metastatic potential. *J. Natl Cancer Inst.*, **85**, 147-152.

NAKAYAMA, T., OHTSURU, A., NAKAO, K., SHIMA, M., NAKATA, K., WATANABE, K., ISHII, N., KIMURA, N. & NAGATAKI, S. (1992). Expression in human hepatocellular carcinoma of nucleoside diphosphate kinase, a homologue of the nm23 gene product. *J. Natl Cancer Inst.*, **84**, 1349-1354.

OKABE-KADO, J., KASUKABE, T., HONMA, Y., HAYASHI, M., HENZEL, W.J. & HOZUMI, M. (1992). Identify of a differentiation inhibiting factor for mouse myeloid leukemia cells with nm23/nucleoside diphosphate kinase. *Biochem. Biophys. Res. Commun.*, **182**, 987-994.

POSTEL, E.H., BERBERICH, S.J., FLINT, S.J. & FERRONE, C.A. (1993). Human c-*myc* transcription factor PuF identified as nm23-H2 nucleoside diphosphate kinase, a candidate suppressor of tumor metastasis. *Science*, **261**, (5120), 478-80.

ROSENGARD, A.M., KRUTZSCH, H.C., SHEARN, A., BIGGS, J.R., BASRKER, E., MARGULIES, I.M.K., KING, C.R., LIOTTA, L.A. & STEEG, P.S. (1989). Reduced nm23/Awd protein in tumour metastasis and aberrant *Drosophila* development. *Nature*, **342**, 177-180.

ROYDS, J.A., STEPHENSON, T.J., REES, R.C., SHORTHOUSE, A.J. & SILCOCKS, P.B. (1993). Nm23 protein expression in ductal in situ invasive human breast carcinoma. *J. Natl Cancer Inst.*, **85**, 727-731.

SASTRE-GARAU, X., LACOMBE, M.L., JOUVE, M., VERON, M. & MAGDELENAT, H. (1992). Nucleoside diphosphate kinase/nm23 expression in breast cancer: lack of correlation with lymph-node metastases. *Int. J. Cancer*, **50**, 533-538.

SPENCER, C.A. & GROUDINE, M. (1991). Control of c-*myc* regulation in normal and neoplastic cells. *Adv. Cancer Res.*, **56**, 1-48.

STAHL, J.A., LEONE, A., ROSENGARD, A.M., PORTER, L., KING, R.C. & STEEG, P.S. (1991). Identification of a second human nm23 gene, nm23-H2. *Cancer Res.*, **51**, 445-449.

STEEG, P.S., BEVILACQUA, G., KOPPER, I., THORGEIRSSON, U.P., TALMADGE, J.E., LIOTTA, L. & SOBEL, M.E. (1988*a*). Evidence for a novel gene associated with low tumor metastatic potential. *J. Natl Cancer Inst.*, **80**, 200-204.

STEEG, P.S., BEVILACQUA, G., POZZATTI, R., LIOTTA, L. & SOBEL, M.E. (1988*b*). Altered expression of nm23, a gene associated with low tumor metastatic potential, during adenovirus 2 Ela inhibition of experimental metastases. *Cancer Res.*, **48**, 6550-6554.

SUGGS, S.V., WALLACE, B.B., HIROSE, T., KAWASHIMA, E.H. & ITAURA, K. (1981). Use of synthetic olinucleotides as hybridization probes: isolation of cloned CDNA sequences for human $\beta_2$-microglobulin. *Proc. Natl Acad. Sci. USA*, **78**, 6613-6617.

TOKUNAGA, Y., URANO, T., FURUKAWA, K., KONDO, H., KANEMATSU, T. & SHIKU, H. (1993). Reduced expression of nm23-H1, but not nm23-H2, is concordant with the frequency of lymph-node metastases of human breast cancer. *Int. J. Cancer*, **55**, 66-71.

VARESCO, L., CALIGON, M.A., SIMI, P., BLACK, D.M., NARDINI, V., CASARINO, L., ROCHI, M., FERRARA, G., SOLOMON, E. & BEVILACQUA, G. (1992). The nm23 gene maps to human chromosome band 17q22 and shows a restriction fragment length polymorphism with *Bgl*II. *Genes Chrom. Cancer*, **4**, 84-88.

WALLET, V., MUTZEL, R., TROLL, H., BARZA, O., WURSTER, B., VERON, M. & LACOMBE, M.L. (1990). *Dictyostelium* nucleoside diphosphate kinase highly homologous to Nm23 and Awd proteins involved in mammalian tumor metastases and *Drosophila* development. *J. Natl Cancer Inst.*, **82**, 1199-1202.

WANG, L., PATEL, U., GHOSH, L., CHEN, H.C. & BANERJEE, S. (1993). Mutation in the nm23 gene is associated with metastases in colorectal cancer. *Cancer Res.*, **53**, 717-720.

YAMAGUCHI, A., URANO, T., FUSHIDA, S., FURUKAWA, K., NISHIMURA, G., YONEMURA, Y., MIYAZAKI, I., NAKAGAWARA, G. & SHIKU, H. (1993). Inverse association of nm23-H1 expression by colorectal cancer with liver metastasis. *Br. J. Cancer*, **68**, 1020-1024.

ZOU, M., SHI, Y., AL-SEDAIRY, S. & FARID, N.R. (1993). High levels of nm23 gene expression in advanced stage of thyroid carcinomas. *Br. J. Cancer*, **68**, 385-388.